# Effects of transdiagnostic group treatment for sleep disturbances in adult attention-deficit/hyperactivity disorders and autistic spectrum disorder: a pilot study

**DOI:** 10.1007/s41105-021-00351-8

**Published:** 2021-11-10

**Authors:** Miho Ishii, Wakako Ito, Yuki Karube, Yuko Ogawa, Anna Tagawa, Shunta Maeda, Hideki Sato, Toru Takahashi, Natsumi Inomata, Hajime Narisawa, Yoshikazu Takaesu, Koichiro Watanabe, Isa Okajima

**Affiliations:** 1 Department of Neuropsychiatry, School of Medicine, Kyorin University, Tokyo, Japan; 2 Senzoku Stress Coping Support Office, Tokyo, Japan; 3 Institute of Neuropsychiatry, Tokyo, Japan; 4 Department of Clinical Psychology, Faculty of Health and Medical Science, Teikyo Heisei University, Tokyo, Japan; 5 Department of Psycho-Oncology, National Cancer Center Hospital, Tokyo, Japan; 6 Department of Neuropsychiatry, NTT Medical Center Tokyo, Tokyo, Japan; 7 Graduate School of Education, Tohoku University, Miyagi, Japan; 8 Department of Disaster Psychiatry, Fukushima Medical University School of Medicine, Fukushima, Japan; 9 Radiation Medical Science Center for the Fukushima Health Management Survey, Fukushima, Japan; 10 Faculty of Human Sciences, Waseda University, Saitama, Japan; 11 Medical Corporation Johohkai, Tokyo, Japan; 12 Department of Psychology, Aichi Shukutoku University, Aichi, Japan; 13 Department of Neuropsychiatry, Graduate School of Medicine, University of the Ryukyus, Okinawa, Japan; 14 Department of Psychological Counseling, Faculty of Humanities, Tokyo Kasei University, Tokyo, Japan

**Keywords:** Cognitive behavioral therapy, Sleep–wake rhythms, Attention-deficit hyperactivity disorder, Autism spectrum disorder, Anxiety, Attention switching

## Abstract

Although adult patients with attention-deficit hyperactivity disorder (ADHD) and autism spectrum disorder (ASD) often have sleep problems, few studies have verified the effect of a psychological approach specific to sleep–wake rhythms on these sleep disturbances. Therefore, the aim of this pilot study was to develop a trans-diagnostic approach with sleep scheduling and regularity of sleep duration as core modules, and to examine the effect of the intervention in adult ADHD and/or ASD subjects with sleep disturbances. This was a within-group pilot study. Ten patients with adult ADHD and/or ASD with sleep disturbances (10 males, age: 27.4 ± 5.6 years) took part in a 90-min weekly group intervention for 5 weeks. All participants were assessed on scales for sleep complaints, anxiety, depression, and symptoms of ADHD and ASD before and after the intervention, and at 3-month follow-up. The results showed that the intervention significantly improved sleep disturbances at post-intervention (*p* = 0.003, *d* = 1.30, 95% CI 0.31–2.28) and at the 3-month follow-up (*p* = 0.035, *d* = 0.41, 95% CI − 0.48 to 1.30). In addition, attention switching for ASD symptoms was significantly reduced post-intervention (*p* = 0.031, *d* = 1.16, 95% CI 0.19–2.13). This is the first pilot study of a trans-diagnostic group approach for adult ADHD and/or ASD with sleep disturbances. The intervention primarily led to an improvement of sleep disturbances, followed by improvement of disease-specific symptoms in adult subjects with ADHD and ASD.

## Introduction

Patients with attention-deficit hyperactivity disorder (ADHD) and autism spectrum disorder (ASD) often have sleep problems [1, 2]. The prevalence of sleep disorders comorbid with adult ADHD is reported to be 82.6%, and approximately 30% in adults with ASD [2]. In particular, disturbance of the sleep–wake rhythm causes deterioration in quality of life (QoL) [3]. Therefore, it is important to establish an adequate treatment for sleep disturbances in neurodevelopmental disorders, focusing on circadian rhythm sleep–wake disorders, such as irregular sleep–wake rhythm and delayed sleep–wake phase. The mainstream treatment for sleep disturbances is pharmacotherapy [4]. However, the effect size of melatonin treatment for circadian rhythm disorder in adult ASD is smaller than that of non-pharmacological therapy [5]. In adult ADHD, an effect of melatonin treatment on advancing sleep–wake phase has been reported [6]. However, there are few relevant studies and the efficacy of melatonin treatment for adult ADHD with irregular sleep–wake pattern has not been reported. Therefore, non-pharmacological therapy is an important tool for addressing neurodevelopmental disorders with sleep–wake rhythm disturbance.

Recently, it has been suggested that psychosocial intervention is effective and safe for sleep disturbance comorbid with neurodevelopment disorder [7]. In ADHD, a study reported that a behavioral intervention improved insomnia (usual care: 35% vs intervention: 56%) and QoL (Cohen's *d* = 0.39) [8]. Similarly, a behavioral intervention for ASD improved insomnia [5]. In a meta-analysis of behavioral intervention for sleep problems, improvements were reported in sleep habits (decrease of 4.71 points on the Children’s Sleep Habits Questionnaire), total sleep time (increased by 24.41 min), sleep latency (decreased by 18.31 min), and sleep efficiency (increased by 5.59%) [9].

Currently, there are many reports that show cognitive behavioral therapy for insomnia (CBT-I) is highly effective for insomnia and delayed sleep-phase syndrome (DSPS) [10]. Of the many components in CBT-I, the most effective is sleep scheduling, including stimulus control and sleep restriction therapies [11, 12]. In addition, Buysse et al. [13] suggested that regularity of sleep duration is a core dimension of sleep health. In this regard, one of the characteristics of ASD and ADHD is that sleep–wake rhythm is easily disturbed [14]; therefore, sleep scheduling and regularity of sleep duration as a core module would likely be effective for adult ADHD and ASD with sleep disturbances.

However, few studies have verified the effect of a trans-diagnostic approach specific to sleep–wake rhythms on sleep disturbances. Therefore, the aim of this pilot study was to develop a trans-diagnostic approach specific to sleep–wake rhythms and to examine the effect of the intervention in adult ADHD and/or ASD with sleep disturbances.

## Methods

### Participants

**Fig. 1:**
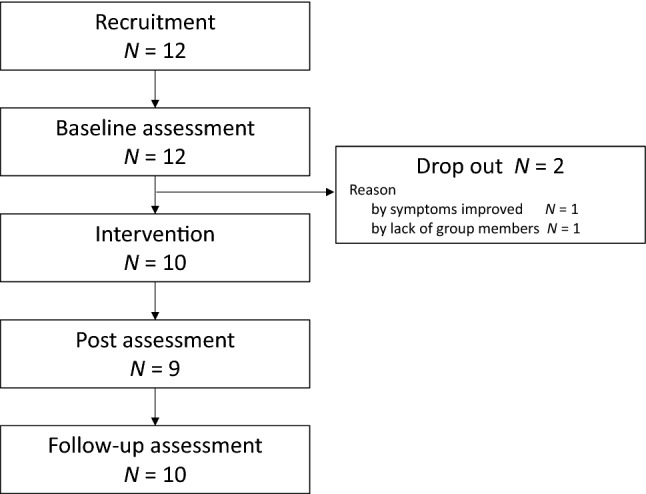
Study flowchart

This study was conducted between September 2017 and June 2018. The participants were recruited through an advertisement placed on a notice board in the Seiwa psychiatric hospital in Japan or were invited to participate by psychiatrists. A total of 12 adult ADHD and/or ASD outpatients with sleep complaints participated in this study. Of these, one showed improvement in their sleep problem before the intervention, and one was unable to participate in the program due to disruption of the sleep–wake rhythm. Therefore, data from 10 participants (10 males, age: 27.4 ± 5.6 years) were analyzed (Fig. 1). The inclusion criteria were as follows: (1) aged 20 years or older; (2) a diagnosis of or suspected ADHD and/or ASD based on the Diagnostic and Statistical Manual of Mental Disorders 5th ed (DSM-5) [15], (3) a score for the insomnia severity index (ISI) ≥ 8-point, (4) having nocturnal sleep problems (i.e., initial, middle, or terminal insomnia) and excessive daytime sleepiness, and (5) taking a stable dose and type of medication for 4 weeks. The exclusion criterion was that their primary disease symptoms were not stable. Two participants were diagnosed with ADHD, seven participants were diagnosed with ASD, and one participant had both ADHD and ASD. Half of the participants were taking hypnotics and 20% of them were taking a psycho stimulant.

### Assessment measures

#### Primary outcome

##### Insomnia severity index (ISI)

This study used the ISI to assess sleep complaints. This self-administered scale consists of seven items that evaluate sleep status for 2 weeks. Each item is scored on a 5-point Likert scale (0–4). The higher the total score (range 0–28), the more severe the insomnia. In this study, a cut-off point for insomnia of ≥ 8 points was utilized [16].

#### Secondary outcomes

##### Hospital anxiety and depression scale (HADS)

To assess depression and anxiety, we used HADS. This self-administered scale consists of seven items for depression and seven items for anxiety. Each item is scored on a 4-point Likert scale (0–3). For each subscale, the higher the total score (range 0–21), the more severe the depression or anxiety [17].

##### Autism-Spectrum quotient (AQ)

We used the AQ to assess ASD symptoms. This self-administered scale consists of five subscales: “Social skill,” “Attention switching,” “Local details,” “Communication,” and “Imagination.” The AQ consists of 50 items, each of which is rated using a 4-point (1–4) Likert scale and subsequently converted to a score in the range 0–1; scores are then summed. The higher the total score, the more severe the ASD symptoms [18].

##### ADHD self-report scale (ASRS)

We used the ASRS to assess ADHD symptoms. This self-administered scale consists of inattentive and hyperactive/impulsivity subscales. The total number of items is 18, and each item is scored on a 5-point (0–4) Likert scale; the higher each subscale’s total points, the more severe the ADHD symptoms [19].

### Intervention

A trans-diagnostic approach, specific to sleep–wake rhythms was used, consisting of weekly 90-min group sessions for 5 weeks. Each group session included two to five participants. The main therapist (I.O.) was a clinical psychologist who has provided CBT for patients with sleep disorders for 10 years or more. Between one and four staff members, who were clinical psychologists, or were qualified to master’s level in clinical psychology, supported the setting of personal sleep schedules and homework assignments.

The intervention content in each session is shown in Table 1. In each session, confirmation of the sleep diary and homework assignments for the last week were first conducted. During the intervention, the sleep schedules and homework assignments for the next week were set for each participant together with the staff. As part of setting homework assignments, they shared homework achievements and new ideas within the groups to expand the repertoire of sleep-promoted behaviors. The personalized homework assignments and sleep schedules, and sharing successful ideas of with other participants were core modules in this intervention. The intervention program consisted of: (1) introduction of the intervention, and how to keep a sleep diary; (2) sleep education (for example, explanations of sleep drive and circadian rhythms); (3) sleep scheduling and regularity of sleep duration; (4) sleep hygiene (for example, naps, core body temperature, and timing of meals), (5) progressive muscle relaxation, and (6) troubleshooting.

**Table 1:** Session content of the intervention

	Contents	Personalized homework assignments
Week 1	Introduction of the intervention program How to keep sleep diary	Sleep diary
Week 2	Confirmation of work on homework assignments for last week Sleep education (light, naps, indoor environment at night) Sleep scheduling	Sleep diary; Setting new time in bed; Setting the timing of bright light exposure; Setting the timing of naps; Setting the control of bright light exposure; Keep regularity of sleep duration
Week 3	Confirmation of work on homework assignments for last week Sleep education (deep body temperature, timing of meals) Adjustment or titration of sleep scheduling	Sleep diary; Setting new time in bed; Setting the timing of meals; Setting the timing of bath; Keep regularity of sleep duration
Week 4	Confirmation of work on homework assignments for last week Sleep education (the relationship between ages and sleep stage) Progressive muscle relaxation Adjustment or titration of sleep scheduling	Sleep diary; Setting new time in bed; Progressive muscle relaxation; Keep regularity of sleep duration
Week 5	Confirmation of work on homework assignments for last week Trouble shooting Adjustment or titration of sleep scheduling	

### Procedure

This study was designed as a pilot study. All participants were assessed for all self-administered scales before and after the intervention, and at 3-month follow-up.

### Data analysis

To confirm the changes in sleep disturbances, total ISI scores were analyzed using a repeated measures one-way analysis of variance (ANOVA) with time (at the pre- and post-intervention and 3-month follow-up) as the independent variable, using the statistical software HAD ver. 17_102 [20]. Then, multiple comparisons were performed using the Holm method. Likewise, the secondary outcomes were analyzed using a repeated-measures ANOVA.

In addition, we estimated the effect sizes of scales within the group using Cohen’s *d* [21]. In general, an absolute *d* value of 0.2 or more indicates a small effect size, *d* of approximately 0.5 indicates a moderate effect size, and *d* of *0*.8 or more indicates a large effect size. The effect sizes of all scales for the group were computed between the scores at pre- vs. post-intervention, between pre-intervention and 3-month follow-up, and between post-intervention and 3-month follow-up.

## Results

Demographic characteristics for the participants are presented in the Table 2, and descriptive statistics of all measures for each group at pre- and post-intervention, and 3-month follow-up are presented in Table 3 and Table 4.

**Table 2:** Participant demographics, clinical profile and medication use at baseline

Characteristics	*N*(%), mean (SD)
Sex	
Female	0 (0)
Male	10 (100)
Age, mean (SD)	27.4 (5.6)
Diagnosis	
ADHD	2 (20)
ASD	7 (70)
Both	1 (10)
Sleep complaint	
Sleep onset latency	3 (30)
Delayed sleep phase	4 (40)
Both	3 (30)
Medication for sleep	5 (50)
Medication for ADHD	2 (20)

*ADHD* attention-deficit hyperactivity disorder, *ASD* autism spectrum disorder, *N* number, *SD* standardized deviation

### Main symptoms

There was a significant effect of time on ISI scores (*F* (2, 16) = 19.34, *p* < 0.001; Tables 3 and 4). Multiple comparisons showed a significant difference between the scores at pre- and post-intervention (*p* = 0.003, *d* = 1.30, 95% CI 0.31–2.28), between scores at pre-intervention and 3-month follow-up (*p* = 0.035, *d* = 0.41, 95% CI − 0.48 to 1.30), and between scores at post-intervention and 3-month follow-up (*p* = 0.002, *d* = − 0.89, 95% CI − 1.82 to 0.04).

**Table 3:** Mean and standard deviation for the outcome measures

	Pre-intervention Mean (SD)	Post-intervention Mean (SD)	3-month-FU Mean (SD)
ISI	16.0 (5.6)	8.8 (4.1)	13.6 (5.0)
HADS			
Depression	9.0 (4.3)	10.1 (3.7)	10.4 (4.3)
Anxiety	11.6 (5.3)	8.1 (3.5)	7.9 (4.9)
ASRS			
Total	56.6 (11.4)	51.7 (9.7)	52.4 (11.2)
Hyperactivity	23.0 (6.0)	19.7 (8.4)	22.5 (6.4)
Inattention	31.4 (6.0)	26.8 (11.1)	29.9 (7.3)
AQ			
Total	31.0 (6.8)	29.4 (7.9)	31.0 (7.4)
Social skill	5.6 (3.2)	5.4 (3.6)	5.2 (3.7)
Attention switching	7.2 (1.0)	5.3 (1.7)	6.7 (1.6)
Local details	5.7 (1.9)	6.3 (2.8)	6.5 (2.6)
Communication	6.7 (1.8)	6.9 (2.7)	6.9 (2.3)
Imagination	5.8 (2.3)	5.4 (2.3)	5.7 (2.1)

*AQ* Autism-Spectrum Quotient, *ASRS* adult ADHD Self-Report Scale, *HADS* Hospital Anxiety and Depression Scale, *ISI* Insomnia Severity Index, *SD* standardized deviation

### Secondary symptoms

The score for HADS-depression (*F* (2, 12) = 0.34, *p* = 0.74) was not significantly affected by time. The score for HADS-anxiety was also not significantly affected by time (*F* (2, 12) = 3.84, *p* = 0.051), but there was moderate effect size between the scores at pre- and post-intervention (*d* = 0.56, 95% CI − 0.45 to 1.56) and between the scores at pre-intervention and 3-month follow-up (*d* = 0.76, 95% CI − 0.27 to 1.78; Tables 3 and 4).

The total ASRS score was not significantly affected by time (*F* (2, 18) = 1.50, *p* = 0.25) but a moderate effect size (*d* = 0.54, 95% CI − 0.36 to 1.44) was seen between the scores at pre-intervention and follow-up (Table 4). Hyperactivity (*F* (2, 18) = 0.89, *p* = 0.43) and inattention subscales (*F* (2, 18) = 0.73, *p* = 0.50) were not significantly affected by time (Tables 3 and 4).

**Table 4:** Effect sizes for each outcome measure

	Effect Size *d* (95% Cl)			*F* value	*p*-value
	Pre-post	Pre-FU	Post-FU		
ISI	1.30 (0.31, 2.28)	0.41 (– 0.48, 1.30)	– 0.89 (– 1.82, 0.04)	19.34	** < 0.001**
HADS					
Depression	– 0.44 (– 1.34, 0.45)	– 0.31 (– 1.20, 0.58)	0.15 (– 0.73, 1.02)	0.34	0.740
Anxiety	0.56 (– 0.45, 1.56)	0.76 (– 0.27, 1.78)	0.29 (– 0.67, 1.27)	3.84	0.051
ASRS					
Total	0.16 (– 0.72, 1.05)	0.54 (– 0.36, 1.44)	0.38 (– 0.51, 1.27)	1.50	0.250
Hyperactivity	0.43 (– 0.42, 1.29)	0.08 (– 0.76, 0.92)	– 0.36 (– 1.29, 0.49)	0.89	0.427
Inattention	0.49 (– 0.36, 1.35)	0.22 (– 0.63, 1.06)	– 0.32 (– 1.16, 0.53)	0.73	0.496
AQ					
Total	0.17 (– 0.71, 1.05)	0.00 (– 0.88, 0.88)	– 0.16 (– 1.04, 0.72)	0.83	0.453
Social skill	0.03 (– 0.85, 0.91)	0.12 (– 0.76, 0.10)	0.08 (– 0.80, 0.97)	0.44	0.655
Attention switching	1.16 (0.19, 2.13)	0.33 (– 0.56, 1.22)	– 0.70 (– 1.61, 0.21)	5.98	**0.011**
Local details	– 0.13 (– 1.02, 0.75)	– 0.40 (– 1.29, 0.49)	– 0.20 (– 1.08, 0.68)	1.34	0.290
Communication	– 0.09 (– 0.97, 0.79)	0.00 (– 0.88, 0.88)	0.08 (– 0.80, 0.97)	0.18	0.835
Imagination	0.04 (– 0.83, 0.93)	0.00 (– 0.88, 0.88)	– 0.05 (– 0.93, 0.83)	0.08	0.922

Bold indicates that the *p*-value is less than 0.05

*AQ* Autism-Spectrum Quotient, *ASRS* adult ADHD Self-Report Scale, *HADS* Hospital Anxiety and Depression Scale, *ISI* Insomnia Severity Index, *95% CI* 95% confidential interval

The total AQ score did not vary significantly with time (*F* (2,16) = 0.83, *p* = 0.45). Only the attention switching subscale showed a significant effect of time (*F* (2, 16) = 5.98, *p* = 0.01). Multiple comparisons revealed a significant difference between the pre- and post-intervention scores (*p* = 0.031, *d* = 1.16, 95% CI 0.19–2.13; Tables 3 and 4).

## Discussion

The aim of this pilot study was to develop a trans-diagnostic approach specific to sleep–wake rhythms and to examine the effect of the intervention in adult ADHD and/or ASD with sleep disturbances. The results showed that the intervention improved sleep disturbances and attention switching among ASD symptoms. This suggests that the trans-diagnostic approach improved sleep problems and partially reduced disease-specific symptoms in adult patients with ADHD and/or ASD. To the best of our knowledge, this is the first study to investigate the effects of the cognitive behavioral group approach for adult ADHD and/or ASD with sleep disturbances.

The results showed that the intervention had a short-term effect on sleep disturbance. This was similar to a previous study of behavioral interventions for insomnia comorbid with ADHD and ASD in childhood [5, 7].

Furthermore, a pilot study that verified group CBT-I in adult ADHD with insomnia showed improvement in insomnia symptoms after treatment (Cohen’s *d* = 0.84) [22]. In this study, the effect size (*d* = 1.30) was larger than that in the previous study. Okajima et al. [23] reported that combination therapy of bright light and CBT-I including sleep scheduling was effective for a 31-year-old individual with irregular sleep–wake rhythm disorder. In the current study, the reason for the larger effect could be that sleep pressures are thought to stabilize with sleep scheduling focused on irregular sleep–wake rhythms.

In addition, the staff support for each participant was considered successful. The support staff encouraged participants to make lifestyle changes while considering their developmental characteristics. This personalized approach was highly effective. Okajima et al. [24] compared the effectiveness of a tailored CBT-I application with a standard CBT-I application for workers with insomnia. Only the tailored CBT-I was found to significantly improve insomnia at 1-month follow-up (Hedges’ *g* = − 0.85) and the effect of the intervention was maintained 3 months later [24]. Therefore, the result of our study suggested that the high effect size at the post-intervention was due to personalized intervention.

However, the effects of the intervention on sleep disturbances did not remain at the 3-month follow-up. One of the reasons for this was the low number of sessions. Although we conducted 5 weekly sessions, a previous study of CBT-I for adult ADHD subjects with insomnia conducted 10 sessions [22]. In addition, it has been suggested that in CBT for DSPS, cognitive restructuring is important [25]. Although the most effective number of sessions is four sessions in general CBT-I [26], it may be necessary to increase that number of CBT sessions and add cognitive restructuring for ADHD and/or ASD with sleep disturbances.

The trans-diagnostic group approach we developed included sleep scheduling and regularity of sleep duration as core modules, and sleep hygiene and relaxation as optional modules. A previous study that was similar to this trans-diagnostic intervention [27] included interventions for healthy students (10–18 years old) with an evening chronotype. In their results, the degree of preference for evening chronotype decreased (*d* = − 0.50, 95% CI 1.05–2.97). Therefore, our trans-diagnostic approach would be expected to be efficacious not only for adults with ADHD/ASD, but also for any individuals with sleep complaints.

There was no significant post-intervention difference in depression and anxiety, but the *p*-value for anxiety was 0.051, and the effect sizes were moderate at post-intervention (*d* = 0.56) and at 3-month follow-up (*d* = 0.76). The findings revealed that the trans-diagnostic approach was likely to reduce anxiety symptoms in adults with ADHD/ASD. Although previous studies reported that CBT-I improved not only insomnia symptoms but also depression symptoms [28, 29], there have been no studies of patients with anxiety disorders. In CBT for anxiety, targeting parents of children with ASD, children with ASD improved not only their anxiety symptoms but also sleep problems [30]. This suggests links between insomnia and anxiety in children with ASD, but the process has not been clarified.

It has been reported that CBT for generalized anxiety disorder with extensive worry reduced anxiety symptoms via improvement of insomnia [31]. This suggests that improvement of sleep disturbances would contribute to the reduction of anxiety symptoms in adults with ADHD/ASD.

The intervention moderately improved the attention problems associated with ASD. This finding is consistent with the results of previous studies [32, 33]. Studies have reported that individuals with insomnia are impaired in subjective cognitive function [32, 33]. It has been suggested that sleep deprivation has the greatest effect on attentional function among the other cognitive function categories, such as processing speed, working memory, short-term memory, reasoning, crystallized intelligence, and verbal fluency [34]. In this study, the elimination of sleep loss by the intervention may have led to improvements in cognitive function. In contrast, there was no improvement in attention as assessed by the ASRS. Although the reason for this is unclear, it might be because only 20% of the participants had a diagnosis of ADHD only in this study.

### Limitations

This study had some limitations. First, although this pilot study provides the first evidence of a trans-diagnosis group approach specific to sleep–wake rhythms in adult ADHD/ASD, it had a small sample size and the participants were all men, which limits the generalizability of our findings. Randomized controlled trials with larger sample sizes should be conducted in the future.

Second, scales for sleep–wake rhythms were not used in this study. Those available include the Biological Rhythms Interview of Assessment in Neuropsychiatry (BRIAN), which measures biological rhythm [35], and the Munich Chronotype Questionnaire (MCTQ), which measures habitual sleep schedule during work days and free days [36]. In the future, it is important to assess changes in sleep–wake rhythms using these scales.

Third, the AQ and ASRS were measured as assessment scales for ASD/ADHD symptoms with similar use as previous studies [22]. Although these scales have confirmed reliability and validity [18, 19], self-administered scales for ASD/ADHD symptoms may not be appropriately assessed, because individuals with ASD/ADHD have difficulties in self-monitoring [37]. In the future, it is important to assess changes in ASD/ADHD symptoms using clinical interview assessments, such as structured interviews.

Finally, no objective sleep parameters, such as polysomnography and actigraphy, were measured. To evaluate the improvement of sleep–wake rhythm, it is necessary to measure objective parameters as well as subjective sleep parameters based on self-ratings.

## Conclusion

This is the first pilot study of a trans-diagnostic group approach for adult ADHD and/or ASD with sleep disturbances. The intervention led to improvement of sleep disturbances and anxiety and partial alleviation of symptoms of ASD.
